# p21 Knockdown as a Therapeutic Strategy for Focal Cartilage Injury Repair

**DOI:** 10.1096/fj.202600387RRR

**Published:** 2026-04-20

**Authors:** Leila Larijani, Aria Ahadzadeh Ardebili, Nini Ortiz Vales, Andreas T. Stürmer, Roman J. Krawetz

**Affiliations:** ^1^ McCaig Institute for Bone & Joint Health University of Calgary Calgary Alberta Canada; ^2^ Department of Biomedical Engineering, Schulich School of Engineering University of Calgary Calgary Alberta Canada; ^3^ T‐Technologies Grundlagenforschung e.U Kremsmünster Austria; ^4^ Department of Biochemistry and Molecular Biology, Cumming School of Medicine University of Calgary Calgary Alberta Canada

**Keywords:** cartilage regeneration, CDKN1A, focal cartilage injury, intraarticular injection, lentiviral vectors, p21, senescence, shRNA therapy

## Abstract

Focal articular cartilage defects lack intrinsic regenerative capacity and can progress to osteoarthritis as no effective treatment exists to slow, stop or reverse cartilage degeneration. Cellular senescence, characterized by elevated p21 (CDKN1A) expression, impairs chondrocyte proliferation and cartilage repair. We hypothesized that targeted p21 suppression via lentiviral delivery would enhance cartilage regeneration. Lentiviral shRNA vectors targeting p21 or non‐sense controls with a tdTomato reporter were constructed. In vitro, murine synovial progenitor cells and MC3T3E1 preosteoblasts were transduced and *p21* mRNA was quantified by qRT‐PCR. Cell cycle analysis was performed using flow cytometry. In vivo, immunocompromised (B6.Cg‐Prkdc^scid^/SzJ) and immunocompetent (C57BL6) mice received full‐thickness cartilage defects followed by intraarticular *p21* shRNA or nonsense control injection. Cartilage repair was assessed by Safranin O staining and tissue cytometry quantified lentiviral transduction and p21 knockdown in chondrocytes. In vitro, *p21* shRNA achieved ~80% reduction in *p21* mRNA and increased G2M phase cells. In vivo, *p21* shRNA treatment significantly improved cartilage repair in both strains. Tissue cytometry revealed 90% transduction efficiency with p21^+^ cells reduced from ~90% to ~30%. Spearman correlation showed significant negative correlation between p21 expression and repair outcomes. Hepatic off‐target transduction was minimal in uninjured animals but increased in injured animals, yet morphological hepatotoxicity was not observed. *p21* knockdown via lentiviral shRNA effectively promotes cartilage regeneration, supporting the concept of targeting p21 and/or the p21 pathway as a therapeutic strategy for cartilage repair.

## Graphical abstract

Intraarticular injection of lentiviral p21 shRNA (targeting Cdkn1a) into murine knees with full‐thickness cartilage defects reduces chondrocyte senescence while enhancing matrix repair with minimal hepatic off‐target effects.

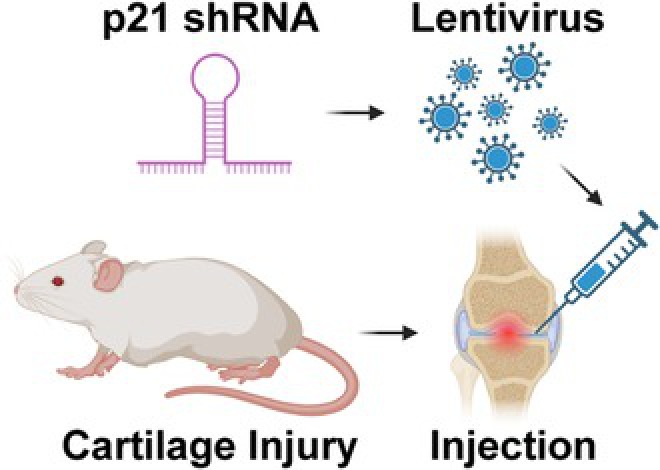

## 1 Introduction

Articular cartilage possesses a limited regenerative capacity that renders focal cartilage defects a significant clinical challenge [1, 2]. The avascular and aneural nature of articular cartilage, combined with the low proliferative capacity of resident chondrocytes, creates an inherent impediment to spontaneous healing following traumatic injury. In the absence of effective interventions, focal cartilage defects often progress to osteoarthritis (OA), leading to chronic pain, reduced joint function, and substantial morbidity [3]. Current therapeutic approaches for cartilage injury, including microfracture, autologous chondrocyte implantation (ACI), and osteochondral grafting, have demonstrated variable efficacy and are frequently limited by anatomical constraints, donor site morbidity, or inadequate tissue quality [4, 5]. Thus, the development of novel biologic strategies capable of promoting chondrogenic regeneration and restoring articular surface integrity remains a critical unmet clinical need.

The pathophysiology of focal cartilage defects extends beyond the initial injury site. Recent evidence demonstrates that untreated focal chondral defects initiate a cascade of progressive degenerative changes in surrounding tissue [6]. Biomechanical studies have shown that focal defects create regions of abnormally elevated strain and contact stress around the defect margin, which drives chondrocyte‐mediated cartilage degradation and matrix loss extending from the defect boundary [7, 8]. This progressive perilesional degeneration is accompanied by activation of the inflammatory cascade, with increased production of proinflammatory cytokines including interleukin‐1*β* (IL‐1*β*), tumor necrosis factor‐*α* (TNF‐*α*), and interleukin‐6 (IL‐6), which stimulate matrix metalloproteinases (MMPs) and further cartilage matrix catabolism [9, 10]. The consequence is that even small, initially contained defects can evolve into large areas of cartilage destruction, underscoring the importance of prompt intervention to halt this degenerative process.

Cellular senescence has emerged as a key pathophysiologic factor limiting both endogenous and therapeutic cartilage repair capacity [6, 10]. Senescent chondrocytes are characterized by elevated expression of cyclin‐dependent kinase inhibitors, particularly p21 (CDKN1A) and p16, which enforce irreversible cell cycle arrest and promote a proinflammatory senescence‐associated secretory phenotype (SASP) [11]. The accumulation of senescent cells in injured cartilage tissue, a hallmark of aging cartilage and post‐traumatic joint degeneration, impairs the proliferative and matrix‐synthesizing capacity of both resident chondrocytes and progenitor cell populations, thereby impairing the natural reparative response [12]. Furthermore, the SASP contributes to chronic joint inflammation through sustained production of IL‐1*β*, TNF‐*α*, and other inflammatory mediators, perpetuating a vicious cycle of progressive cartilage degeneration [13]. Given the prominent role of senescence in cartilage pathology, strategies aimed at modulating senescence‐related pathways represent a promising therapeutic avenue.

p21 has been identified as a key regulatory node in the control of both cell proliferation and cellular senescence. As a cyclin‐dependent kinase inhibitor, p21 mediates cell cycle arrest through inhibition of cyclin E‐CDK2 and cyclin A‐CDK2 complexes, thereby preventing phosphorylation of the retinoblastoma protein and blocking G1/S phase transition [14]. However, accumulating evidence indicates that p21 expression is negatively associated with chondrogenic differentiation capacity and cartilage regenerative potential [15, 16, 17, 18]. During normal chondrogenesis, a transient period of cell cycle withdrawal precedes robust extracellular matrix (ECM) synthesis; however, excessive or prolonged p21 expression impairs the delicate balance between proliferation and differentiation required for effective cartilage regeneration [19]. In mesenchymal stem cells (MSCs) and induced pluripotent stem cells (iPSCs), reduction of p21 expression through genetic deletion or shRNA‐mediated knockdown has been shown to substantially enhance both proliferative capacity and chondrogenic differentiation potential while preserving Type II collagen (COL2A1) synthesis and glycosaminoglycan (GAG) deposition [17, 18, 20, 21]. Importantly, p21 knockdown in iPSC‐derived chondrocytes enabled extensive cell expansion, approximately 150‐fold over three additional passages, without loss of subsequent GAG production, whereas control cells showed dramatically reduced matrix synthesis capacity with serial passaging. In vivo studies using p21‐deficient iPSCs have demonstrated superior cartilage regeneration compared to wildtype controls in murine full‐thickness cartilage defect models, with enhanced Type II collagen deposition and reduced fibrocartilage formation [15, 16]. Additionally, pharmacological inhibition of p21 in vivo has been shown to enhance chondrocyte proliferation while maintaining robust Type II collagen expression, with Type II collagen‐positive chondrocytes remaining proliferative following p21 suppression [22]. These findings suggest that targeted suppression of p21 may represent an effective strategy to overcome the regenerative limitations of injured cartilage by simultaneously promoting chondrocyte proliferation and maintaining chondrogenic commitment.

RNA interference (RNAi)‐based therapeutics have emerged as powerful tools for targeted gene knockdown in tissue engineering and regenerative medicine applications. shRNA and small interfering RNA (siRNA) technologies enable selective suppression of specific genes with high target specificity and potency, allowing for local modulation of disease‐relevant pathways [23]. Furthermore, lentiviral‐based shRNA vectors offer the advantages of stable, long‐term gene knockdown through chromosomal integration, enabling sustained suppression of target genes throughout the repair process [24]. Intraarticular injection provides a targeted, local therapeutic approach that minimizes systemic exposure and potential off‐target effects while delivering therapeutic agents directly to the injured tissue microenvironment.

The objective of the present study was to evaluate the efficacy of *p21* shRNA delivered via intraarticular injection in promoting cartilage regeneration in a murine focal cartilage defect model. We hypothesized that local suppression of p21 expression would enhance chondrocyte proliferation and chondrogenic differentiation, leading to improved repair of focal cartilage injuries and restoration of articular surface integrity.

## 2 Methods

### 2.1 p21 shRNA Production and Lentivirus Packaging

Two *p21* shRNAs and one Control (nonsense vector shRNA) were used in this study. The p21 shRNA plasmids used in this study were acquired from Addgene (plasmids # 25868, # 25869). The respective p21 shRNAs were then subcloned into pLV[Exp]‐H1 (VectorBuilder) with the addition of a tdTomato ORF driven by the CAG promoter. The resulting lentivirus was produced, purified, and characterized by VectorBuilder.

*shRNA injection*: Three days post‐injury, mice received intra‐articular injections of 1 × 10^8^ viral particles in 2 μL phosphate‐buffered saline (PBS). Mice were euthanized 4 weeks post‐injury.

### 2.2 p21 shRNA in Vitro Validation

To obtain primary murine synovial cells [25], synovial tissue was dissected from the joint and placed in PBS with 1% antibiotic‐antimycotic (Life Technologies). Grooves were made in the bottom of a 12‐well plate with a scalpel to enhance tissue adherence. Tissue samples were transferred to the plate and cultured in MSC expansion medium consisting of DMEM/F‐12 (Biowhittaker), 10% fetal bovine serum, 1% nonessential amino acids, 1% anti‐anti, and 0.2% *β*‐mercaptoethanol (Life Technologies). Cultures were maintained at 37°C, 2% O₂, and 5% CO_2_. Medium was changed every 2–3 days until cell outgrowth occurred and then daily. Upon reaching confluence, tissue remnants were removed, and cells were passaged into T25 flasks. To select for Sca1^+^ progenitors, the cell suspension was resuspended in diluted BD IMAG buffer (1 mL 10× buffer + 9 mL water) kept on ice. Five microliters of BD immune cell depletion cocktail was added and incubated on ice for 15 min, followed by 5 μL magnetic particles (BD). Samples were placed in a sorting magnet for 7 min, liquid removed, and the procedure repeated using a Sca1 biotinylated antibody (eBioscience) for positive selection. Chondrogenic differentiation was achieved by seeding 10 000 cells/well into a 24‐well plate via hanging drop. The following day, 1 mL of chondrogenic medium was added, and cells were cultured at 37°C in 2% O_2_ for 21 days with medium changes every 2–3 days.

MC3T3‐E1 preosteoblasts (subclone 4, ATCC CRL‐2593) were maintained in *α*‐minimum essential medium (*α*‐MEM; Gibco) supplemented with 10% fetal bovine serum and 1% penicillin–streptomycin at 37°C in a humidified atmosphere of 5% CO_2_.

Synovial progenitors and MC3T3‐E1 cells were seeded in six‐well plates at 1.5–2.0 × 10^5^ cells/well in complete medium and infected the following day at 50%–70% confluence with p21 shRNA or nonsense lentivirus at a multiplicity of infection (MOI) of 5–10 in the presence of 8 μg/mL polybrene, with gentle rocking of plates to ensure even viral distribution; after 16–24 h, viral supernatant was replaced with fresh complete medium and cells were allowed to recover for an additional 48 h. At 72 h postinfection, cells were detached with 0.05% trypsin–EDTA, washed, and resuspended in icecold phosphate‐buffered saline (PBS) containing 2% FBS and 1 mM EDTA, passed through a 40 μm cell strainer to obtain a single‐cell suspension, and subjected to fluorescence‐activated cell sorting (FACS) on a BD FACSAria or equivalent cell sorter to isolate tdTomato positive (transduced) cells, using nontransduced Synovial progenitors or MC3T3‐E1 cells as a negative fluorescence control and gating to exclude debris and doublets. Sorted tdTomato^+^ p21 shRNA and tdTomato^+^ nonsense shRNA populations were collected into tubes containing DPBS, centrifuged, resuspended in TRIZOL for mRNA analysis.

### 2.3 Quantitative PCR Analysis

Total RNA was isolated from sorted tdTomato^+^ Synovial progenitors or MC3T3‐E1 cells using TRIzol reagent (Invitrogen) according to the manufacturer's protocol, with an additional chloroform extraction and isopropanol precipitation step to maximize yield and purity; RNA concentration was assessed by NanoDrop spectrophotometry (A260/280 ratio > 1.9). Complementary DNA (cDNA) was synthesized from 1 μg total RNA using the High‐Capacity cDNA Reverse Transcription Kit (Applied Biosystems) in a 20 μL reaction volume containing random hexamers, following the standard thermal cycling protocol (10 min at 25°C, 120 min at 37°C, 5 min at 85°C). Quantitative real‐time PCR (qRT‐PCR) was performed on the Applied Biosystems QuantStudio 6 Flex Real‐Time PCR System using Taqman probes in 10 μL reactions. Relative gene expression (*Cdkn1a*: Mm04207341m1, *Col2A1*: Mm01309565m1, *Sox9*: Mm00448840m1, *Col10a1*: Mm00487041m1) was calculated using the ΔΔCt method with 18 s rRNA (Hs99999901s1) as the endogenous reference gene and scrambled shRNA samples as calibrator, where fold change = 2^(−ΔΔCt)^; all samples were run in technical triplicates, and experiments were performed with at least three biological replicates, with statistical significance determined by unpaired two‐tailed Student's *t*‐test (*p* < 0.05).

### 2.4 In Vivo Full‐Thickness Cartilage Defect Model

All procedures were approved by the University of Calgary Animal Care Committee (AC20‐0042) and performed in accordance with Canadian Council on Animal Care guidelines.

*Surgical procedure*: A total of 10 mice (5 male and 5 female) were used per treated group in this study. Immunocompromised (B6.Cg‐Prkdc^scid^/SzJ) or immunocompetent (C57BL/6) mice received 0.1 mL buprenorphine (subcutaneous) and 0.04 mL bupivacaine at the surgical site. Under isoflurane anesthesia (5% induction, 1.5% maintenance), a 5 mm incision was made on the medial knee. The patella was displaced, and a 0.6 mm diameter full‐thickness defect was created in the femoral groove using a 26‐gauge needle with twisting motion, penetrating subchondral bone (confirmed by blood appearance) [15, 25, 26]. The patella was repositioned, and skin was closed with sterile wound clips. Post‐operative buprenorphine (0.1 mL) was administered every 12 h for 72 h. To minimize the risk of post‐surgical infection, all surgeries were performed under aseptic conditions in a dedicated surgical space with dedicated surgical tools.

### 2.5 Histology and Immunofluorescence

Knee joints were fixed in 10% neutral buffered formalin for 7 days at room temperature with gentle agitation, followed by decalcification in 10% EDTA solution for 14 days, with regular solution changes to ensure complete mineral removal. Following decalcification, specimens were processed through graded alcohols and xylene and embedded in paraffin. Sagittal sections (10 μm) were cut using a microtome, and approximately 60 sections per sample were collected, of which ~10 encompassed the defect region based on anatomical landmarks.

For histological assessment of cartilage repair, sections were deparaffinized, rehydrated, and stained with Safranin O/Fast Green to visualize proteoglycan content and overall tissue architecture. An established scoring system was used to evaluate cell morphology (0–4), matrix staining (0–3), surface regularity (0–3), cartilage thickness (0–2), and integration with native cartilage (0–2), with a maximum score of 14 for intact native cartilage and 0 indicating a complete defect [27, 28]. Scoring was performed on multiple sections per joint by observers trained in the scoring system, with blinding to treatment group where possible.

For immunofluorescence, sections underwent antigen retrieval in 10 mM sodium citrate buffer, followed by blocking with goat serum to reduce nonspecific binding. Sections were then incubated with primary antibodies specific for tdTomato (Origene: AB8181), p21 (Biolegend: 645951), p16 (Cell Signaling: 23200), Col2 (DSHB: II‐II6B3), IL‐6 (DSHB: CPTC‐IL6‐1). Primary antibodies were conjugated to fluorophores (ABCAM Lightning Kits) or standard fluorophore‐conjugated secondary detection were applied as required. Nuclei were counterstained with DAPI using Everbrite Hardset mounting medium, and slides were imaged on an Axio Scan.Z1 platform. Image analysis and tissue cytometry were used to quantify the proportion and localization of tdTomato and p21‐positive cells within the cartilage defect.

### 2.6 Tissue Cytometry

To quantify fluorescent cell populations and assess transfection efficiency, digital whole‐slide scanning was performed using TissueGnostics StrataQuest software (version 8.0) [29, 30], a contextual tissue cytometry platform that enables observer‐independent quantification of fluorescently labeled cells. The analysis workflow incorporated the following steps [1]: automated nuclei segmentation using the deep learning‐based nuclear segmentation engine to identify individual cell nuclei based on DAPI counterstaining; [2] measurement of fluorescent marker expression within defined nuclear and cytoplasmic compartments for each segmented cell [3]; definition of cellular phenotypes using Boolean gating logic to identify p21‐shRNA transduced cells (positive for tdTomato) and target markers of interest (p21, p16, IL‐6); and [4] spatial context analysis to quantify cell phenotypes within distinct tissue regions. The software generated comprehensive data outputs including absolute cell counts, percentage of fluorescent‐positive cells within each tissue region, mean fluorescence intensity (MFI), and spatial distribution maps. All image analysis was performed in a blinded manner, with analysis templates established on representative samples and applied uniformly across all experimental groups.

### 2.7 Statistical Analysis

All quantitative data were analyzed using GraphPad Prism (version 10.0). Normality was assessed using the D'Agostino‐Pearson test, and homogeneity of variance was evaluated with Levene's test. Group comparisons between control and p21‐shRNA treatment were performed using unpaired *t*‐tests. Fluorescent cell quantification was analyzed using one‐ or two‐way ANOVA where appropriate. All analyses employed *α* = 0.05 as the significance threshold.

## 3 Results

### 3.1 Validation of p21 Knockdown

**FIGURE 1:**
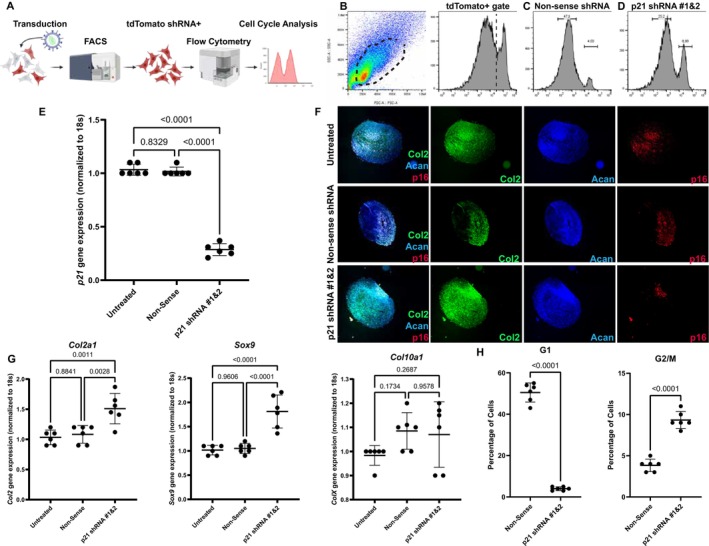
Validation of *p21* knockdown in murine synovial progenitors. Flowchart of the experimental design (A). FACS was employed to identify and purify cells expressing tdTomato (B). Cell cycle analysis was undertaken by flow cytometry, and the percentage of cells within G1 and G2/M are shown (C, D). qPCR was performed on the tdTomato positive cells expressing the nonsense or *p21* shRNA (E). Chondrogenic differentiation was undertaken on the untreated, nonsense, and p21 shRNA (1&2) cell populations. The pellets were sectioned and stained for Collagen 2 (Col2), Aggrecan (Acan), and p16 (F). qPCR was performed on the resultant chondrogenic pellets for *Col2a1*, *Sox9*, and *Col10a1* (G). The percentage of cells in G1 and G2/M were quantified (H). Six technical replicates were used per group, and one‐way ANOVA or *t*‐test was used to determine significance. *p* was set to 0.05.

Murine synovial progenitor cells were isolated from C57BL/6 mice as previously described [25]. The cells were transduced with lentiviral particles containing nonsense, or the combination of two different *p21* shRNA (Figure 1A). The shRNA vector contained a tdTomato reporter and there the tdTomato+ population was purified by FACS (Figure 1B). Cell cycle analysis was undertaken using flow cytometry in the nonsense and p21 shRNA treated cells (Figure 1C,D). Approx. ~4% of the cells were identified in G2/M in the nonsense treated cells and this increased to ~10% with the combination of both p21 shRNAs (Figure 1C,D,H). The tdTomato^+^ cells from each treatment group were also processed for mRNA extraction and the level of *p21* mRNA was quantified (Figure 1E) using 18 s as a housekeeping gene. Murine synovial progenitor cells transduced with the nonsense shRNA showed no difference in *p21* expression vs. the untreated cells, while the cells treated with both *p21* shRNA together demonstrated an approx. 75% reduction of *p21* expression (Figure 1E). The cells were then assayed for their chondrogenic potential using immunofluorescence (Figure 1F) and qPCR as outcome measures (Figure 1G). All chondrogenic pellets expressed Col2 and Acan; however, there was a noticeable decrease in the expression of the senescence marker p16 in cells treated with both p21 shRNAs (Figure 1F). There was also an increase in Col2a1 and Sox9 gene expression in the cells treated with both p21 shRNAs vs. the other groups, but no differences were observed in Col10a1 gene expression (Figure 1G) between groups.

Since it was difficult to obtain a sufficient number of primary synovial progenitor cells to examine both p21 shRNA molecules alone, murine MC3T3‐E1 cells were also employed to validate the efficacy of the p21 shRNAs (Figure S1). Murine MC3T3‐E1 cells were transduced with lentiviral particles containing: nonsense, two different *p21* shRNA, or the combination of both *p21* shRNA (Figure S1A). Since the vector contained a tdTomato reporter, the transduced cells were purified by FACS for the tdTomato^+^ fraction (Figure S1B–D). The tdTomato^+^ cells from each treatment group were then processed for mRNA extraction and the level of *p21* mRNA was quantified (Figure S1E) using 18 s as a housekeeping gene. MC3T3‐E1 cells transduced with the nonsense shRNA showed no difference in *p21* expression vs. the untreated cells, while the cells treated with *p21* shRNA 1, *p21* shRNA 2, or the addition of both *p21* shRNA together demonstrated an approx. 80% reduction of *p21* expression (Figure S1E). To determine if this decrease in endogenous p21 levels had a functional outcome on the cells, cell cycle analysis was performed using flow cytometry. It has previously been shown that reduced p21 expression and/or activity is typically accompanied by an accumulation of cells in G2/M [31]. In the untreated and nonsense shRNA treated cells, approx. ~15% of the cells were identified in G2/M (Figure S1F–G). When *p21* expression was knocked down with shRNA 1, 2, or the combination of both, there was a significant increase in the number of cells observed in G2/M (Figure S1K) with a corresponding decrease of cells in G1 (Figure S1J). It was also observed that the combination of *p21* shRNA 1 and 2 resulted in a significant increase in cells in G2/M vs. either shRNA alone; therefore, the combination of both *p21* shRNAs was used for all in vivo experiments.

### 3.2 Cartilage Injury Repair in Immunocompromised Mice

**FIGURE 2:**
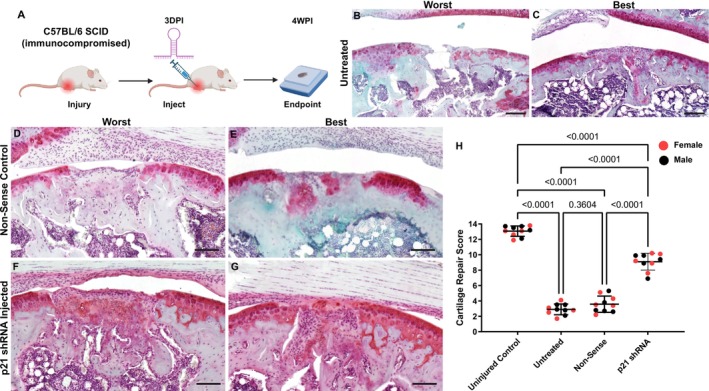
Cartilage repair in immunocompromised mice treated with lentivirus containing *p21* shRNA. Flowchart of the experimental design (A). Histological sections stained with Safranin O centered on the cartilage injury, showing the worst and best outcomes in each group: Untreated (B, C), nonsense shRNA (D, E), and *p21* shRNA (F, G). The cartilage repair score was quantified (E). Ten biological/mice replicates were used per group (5 M, 5F), and one‐way ANOVA was used to determine significance. *p* was set to 0.05. Scale bars equal 25 μm.

Since it has been previously demonstrated that mice can have an immunological response to lentivirus [32], the first set of in vivo experiments was conducted in B6.Cg‐Prkdc^scid^/SzJ mice to avoid this potential confounding variable (Figure 2). Mice received an intraarticular injection of non‐sense or *p21* shRNA 3 days postinjury (3DPI) and were sacrificed at 4 weeks postinjury (4WPI). The knee joints were processed for histological staining and cartilage grading (Figure 2A). The best and worst outcomes for the untreated, nonsense, and *p21* shRNA treatment groups are shown (Figure 2B–G). The untreated mice or those that received the nonsense shRNA demonstrated little to no cartilage repair postinjury (Figure 2B–E), while the mice that received the combination of *p21* shRNA's 1&2 demonstrated moderate cartilage repair, highlighted by the presence of Safranin O staining within the injury site (Figure 2F,G). The repair was quantified, and while no difference was observed between the untreated and nonsense groups, a significant increase in cartilage repair was observed in the *p21* shRNA treatment group (Figure 2H).

### 3.3 Cartilage Injury Repair in Immunocompetent Mice

**FIGURE 3:**
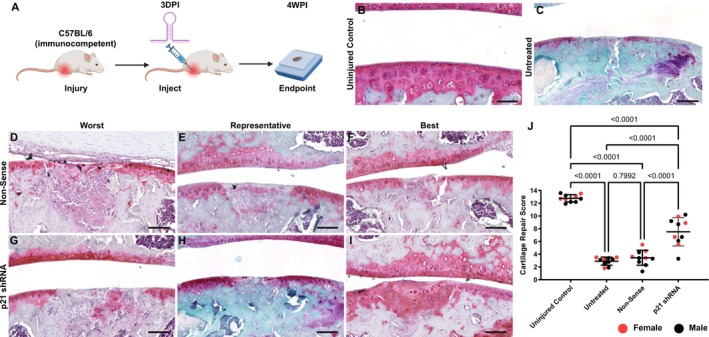
Cartilage repair in immunocompetent mice treated with lentivirus containing *p21* shRNA. Flowchart of the experimental design (A). Histological sections stained with Safranin O centered on the area where the cartilage injury was generated (B), postinjury without treatment (C) and showing the worst, best, and representative outcomes in each treatment group: Non‐sense shRNA (D–F) and *p21* shRNA (G, I). The cartilage repair score was quantified (J). Ten biological/mice replicates were used per group (5 M, 5F) and one‐way ANOVA was used to determine significance. *p* was set to 0.05. Scale bars equal 25 μm.

To determine whether there was any change of efficacy with the lentivirus delivery in animals with an intact immune system, the experiment was repeated with C57BL/6 immunocompetent mice (Figure 3 **A**). As done previously, mice received an intraarticular injection of non‐sense or *p21* shRNA 3 days postinjury (3DPI) and were sacrificed at 4 weeks postinjury (4WPI). The knee joints were processed for histological staining and cartilage grading (Figure 3B–I). Uninjured cartilage (Figure 3B) and an example of an untreated cartilage injury (Figure 3C) show the lack of endogenous repair in C57BL/6 mice. The best, worst and representative outcomes for nonsense (Figure 3D–F) and *p21* shRNA (Figure 3G–I) treatment groups are shown. The mice that received the nonsense shRNA demonstrated little to no cartilage repair post‐injury (Figure 3D–F), while the mice that received the combination of *p21* shRNAs 1&2 demonstrated moderate cartilage repair, highlighted by the presence of Safranin O staining within the injury site (Figure 3G–I). The repair was quantified, and while no difference was observed between the untreated and nonsense groups, a significant increase in cartilage repair was observed in the *p21* shRNA treatment group (Figure 3J).

To better characterize the cartilage repair at the molecular level, staining for the cartilage ECM marker Col2, the senescence marker p16 and the proinflammatory marker IL‐6 was undertaken (Figure S2). In uninjured cartilage, robust Col2 staining can be observed within the tissue, with little p16 and IL‐6 staining present (Figure S2A). In mice that underwent cartilage injury and treatment with the nonsense shRNA, the Col2 staining is disrupted within the injury site and both p16 and IL‐6 staining were observed (Figure S2A). In mice receiving the p21 shRNA, there appears to be some improvement in the Col2 staining and little to no p16 staining observed. However, IL‐6 staining was still observed within the injury site (Figure S2A). The mean fluorescent intensity (MFI) of Col2 in addition to the number of p16 and IL‐6 positive cells were quantified in the injury site (Figure S2B–D). There was a significant increase in Col2 MFI in the mice that received the p21 shRNA, coupled with a significant decrease in p16 positive cells in the same treatment group. There was no difference in IL‐6 positive cells between nonsense vs. p21 shRNA treatment groups, and both were significantly elevated compared with the uninjured group (Figure S2D).

### 3.4 Efficiency of p21 Knockdown in Immunocompetent Mice

**FIGURE 4:**
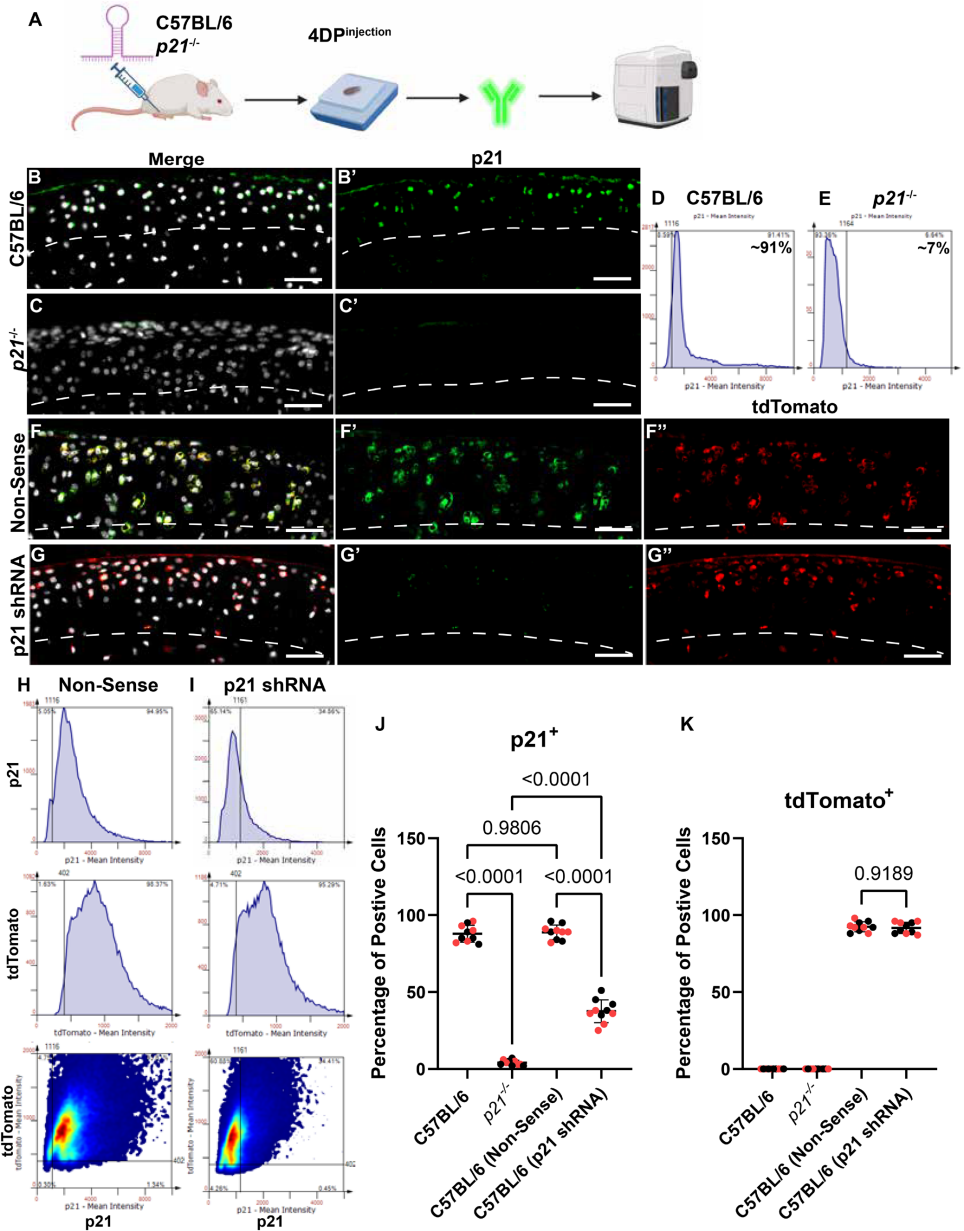
Lentivirus delivery into articular chondrocytes and p21 knockdown. Flowchart of the experimental design (A). Histological sections stained with DAPI, p21 (B′, C′, F′, G′) and tdTomato (F″, G″). Tissue cytometry was used to quantify p21 expression in articular chondrocytes in C57BL/6 (D) and *p21* ^−/−^ (E) mice. Tissue cytometry was also employed to quantify p21 and tdTomato expression in articular chondrocytes of C57BL/6 mice injected with non‐sense (H) or *p21* (I) shRNA. These results were examined statistically with a one‐way ANOVA and p was set to 0.05 (J, I). Ten biological/mice replicates were used per group (5 M, 5F). The dashed line represents the border between the articular cartilage and underlying subchondral bone. Scale bars equal 25 μm.

Since cartilage repair was observed in the C57BL/6 wild‐type mice post‐shRNA treatment, it was decided to better characterize the effectiveness of the lentivirus delivery of the *p21* shRNA into articular chondrocytes and the efficacy of p21 knockdown in those cells (Figure 4A). For this experiment, lentivirus containing *p21* or nonsense shRNA was injected into the uninjured joints of C57BL/6 mice, and the joints were harvested 4 days after injection for histological analysis (Figure 4A). C57BL/6 (Figure 4B) and *p21* ^−/−^ (Figure 4C) mice were used as positive and negative controls, respectively, for p21 expression. Using a tissue cytometry workflow, it was observed that ~90% of articular chondrocytes expressed p21 protein in C57BL/6 mice (Figure 4D), while only ~7% of chondrocytes expressed p21 in *p21* ^−/−^ mice (Figure 4E), which can be attributed to background and/or nonspecific staining. Uninjured C57BL/6 mice were then injected with either nonsense (Figure 4F) or *p21* (Figure 4G) shRNAs. tdTomato expression (shRNA backbone) was detected in ~90% of the articular chondrocytes (Figure 4K), showing that the lentivirus was highly effective at delivering the shRNA to the chondrocytes. The nonsense shRNA had no impact on p21 expression levels, while the *p21* shRNA resulted in only ~30% of chondrocytes still having detectable levels of p21 protein (Figure 4J). Overall, these results show that the lentivirus delivery of the *p21* shRNA into articular chondrocytes and the knockdown of p21 expression in these cells were highly effective.

### 3.5 Correlation Between p21 Expression and Cartilage Repair

**FIGURE 5:**
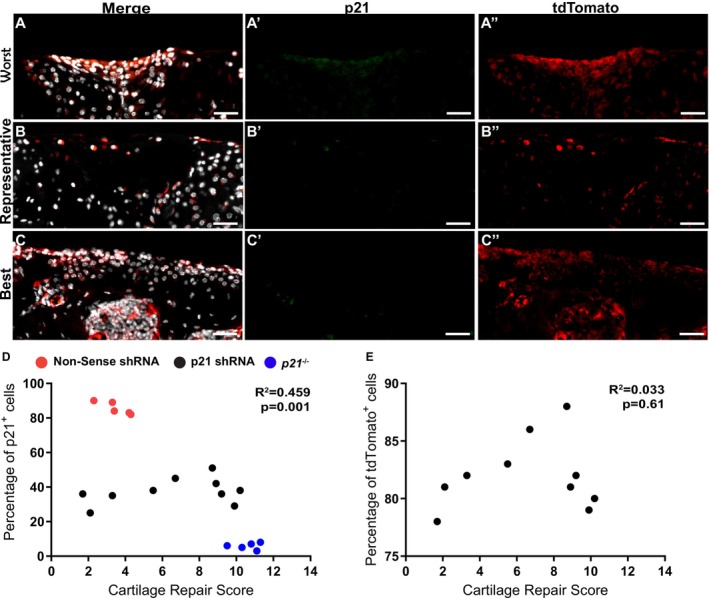
Correlation between cartilage repair a p21 expression. Histological sections from the worst (A), representative (B) and best (C) cartilage repair outcomes in the *p21* shRNA group. These were stained with DAPI, p21 (A′–C′) and tdTomato (A″–C″). A spearman correlation was undertaken between the number of p21 (D) or tdTomato (E) positive cells within the defect site. Nonsense shRNA (*n* = 5), *p21* shRNA (*n* = 10) and *p21* ^−/−^ mice (*n* = 5). Scale bars equal 25 μm.

Since we and others have demonstrated that *p21* ^−/−^ mice have enhanced endogenous cartilage repair [15, 16], it was next examined if there was any relationship between p21 levels and cartilage repair in the mice that received the *p21* shRNA. The cartilage injury site was examined in mice receiving the nonsense shRNA, the *p21* shRNA and *p21* ^−/−^ mice as a control (Figure 5). In the mice receiving the *p21* shRNA, tdTomato expression was observed in the defect site of all mice regardless of the cartilage repair outcome (Figure 5A″–C″). In all the mice that received the p21 shRNA, little to no p21 protein expression was observed (Figure 5A′–C′). The level of p21 expression in articular chondrocytes was correlated with the cartilage outcome and a significant negative correlation was observed between p21 expression and cartilage repair (Figure 5D). An additional correlation was undertaken between tdTomato expression and cartilage repair to determine if the efficiency of the lentivirus transduction might be a confounding variable in this experiment; however, no relationship was observed (Figure 5E).

### 3.6 Systemic Immune Response and Off‐Target Transduction in Liver Tissue

**FIGURE 6:**
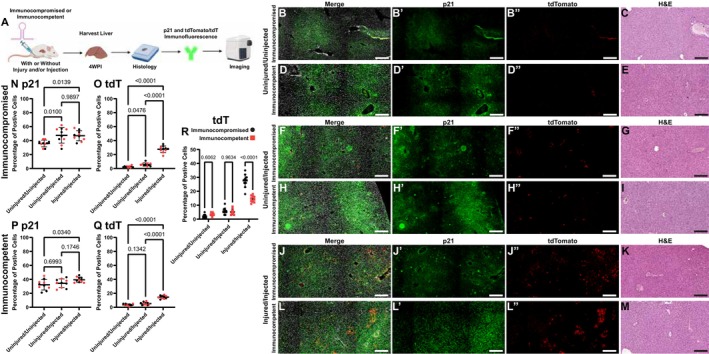
Systemic off‐target transduction following intraarticular lentiviral injection. Experimental design flowchart showing tissue collection timepoint and analysis workflow (A). Immunofluorescence analysis of liver tissue from uninjured, uninjected control mice in immunocompromised (B, C) and immunocompetent (D, E) groups, showing minimal background tdTomato signal and baseline p21 expression. Liver tissue from uninjured, injected immunocompromised (F, G) and immunocompetent (H, I) mice demonstrating tdTomato and p21 expression. Representative liver tissue from injured, injected immunocompromised (J, K) and immunocompetent (L, M) mice showing off‐target transduction. Right panels show corresponding hematoxylin and eosin (H&E) staining demonstrating normal liver parenchymal architecture across all treatment groups with no evidence of hepatic inflammation or tissue damage. Quantitative tissue cytometry analysis of p21 (N, P) and tdTomato (O, Q) positive cell populations in liver tissue. Quantification of tdTomato‐positive cell populations across all groups (R). Statistical analysis was performed using one‐way (N–Q) or two‐way ANOVA (R) with *p* values indicated above bracketed comparisons. Scale bars equal 25 μm for all panels. *n* = 10 mice per group (5 male, 5 female). Data represent mean ± standard deviation of tissue cytometry quantification.

To assess potential off‐target transduction and systemic immune responses to the lentiviral vectors, liver tissue was harvested from both immunocompromised and immunocompetent mice at 4 weeks postinjection (in injured and non‐injured mice) (Figure 6A) [33]. Representative immunofluorescence images demonstrated minimal background staining in the tdTomato channel from livers of uninjected mice (Figure 6B,D). In uninjured mice receiving *p21* shRNA injections into the joint, minimal tdTomato signal was detected in the liver, indicating that off‐target transduction of hepatic cells by the lentiviral vectors did occur, but was minimal in both the immunocompromised and immunocompetent groups (Figure 6F,H). In contrast, mice that had cartilage injuries and received *p21* shRNA showed moderate tdTomato expression in the liver, suggesting some degree of systemic circulating lentiviral particles or transduced cells migrating to distal sites in both the immunocompromised and immunocompetent groups (Figure 6J,L). The tissues from all mice were also stained for p21 expression and mice within all treatment groups exhibited p21 positive staining throughout the liver (Figure 6B–L). It was noted that cells positive for tdTomato (red) in the liver also presented with diminished p21 staining (green) (Figure 6J,L), suggesting successful knockdown of p21 in transduced hepatic cells. However, a knockdown of p21 expression in the liver as a whole was not achieved; instead, an increase in p21 expression was observed in injected mice vs. the uninjected controls (Figure 6N). Another interesting finding was that in both immunocompromised and immunocompetent mice that underwent cartilage injury, there was a significant increase in the number of p21 positive cells in the liver (Figure 6N,P). Quantification of tdTomato‐positive cells in the livers of injected/uninjured and injected injured mice (immunocompromised and immunocompetent) showed a significant increase in infectivity of the hepatic cells with cartilage injury (Figure 6O,Q). Furthermore, a direct comparison between the immunocompromised vs. immunocompetent injured groups shows a dramatic increase in infectivity in immunocompromised mice that received the injury (Figure 6R). Histological assessment of liver parenchyma using hematoxylin and eosin (H&E) staining revealed normal hepatic architecture in all groups, with no evidence of inflammatory infiltration, hepatocellular necrosis, or tissue damage associated with lentiviral transduction or p21 knockdown (Figure 6C,E,G,I,K,M). This indicates that despite detectable systemic transduction following injury, the lentiviral delivery system did not induce overt hepatotoxicity or immune‐mediated pathological responses in either immunocompromised or immunocompetent mice.

## 4 Discussion

This study demonstrated that targeted p21 suppression via lentiviral shRNA delivery promotes cartilage regeneration following focal injury. The robust efficacy across both immunocompromised and immunocompetent models establishes p21 suppression as a viable therapeutic strategy and advances senescence‐targeted regenerative medicine.

### 4.1 p21 as a Regulator of Chondrocyte Proliferation and Repair

The ~80% reduction in p21 mRNA expression, coupled with increased G2M phase accumulation (~45% vs. ~15% in controls), confirms p21's role as a potent cell cycle inhibitor. The superior effects of combined *p21* shRNA (~45% G2M) compared to individual constructs (~30% G2M) suggest synergistic pathway suppression. In vivo, 90% lentiviral transduction efficiency in articular chondrocytes with p21 knockdown reducing p21^+^ cells from 90% to 30% demonstrates high specificity and efficacy. Importantly, meaningful cartilage repair was achieved despite incomplete p21 knockdown, indicating that substantial reduction rather than complete elimination is therapeutically sufficient. This partial knockdown phenotype is advantageous from a safety standpoint, as complete p21 ablation could theoretically impair critical tumor‐suppressor functions.

The significant negative correlation between p21 expression and repair outcomes provides mechanistic validation that p21 abundance directly influences regenerative potential. Critically, this correlation was independent of transduction efficiency, indicating that superior repair outcomes reflect the functional consequence of reduced p21 expression rather than higher transduction rates.

### 4.2 Mechanisms and Clinical Significance

p21 reduction may promote repair through multiple mechanisms. Reduced p21 can permit chondrocytes to pass the G1/S checkpoint and re‐enter the cell cycle, potentially supporting expansion of the resident chondrocyte population. At the same time, preserved Safranin O staining in treated animals suggests that p21 suppression does not compromise the chondrogenic phenotype, raising the possibility that both proliferation and differentiation programs may be maintained. Beyond cell cycle effects, diminished p21 expression could modulate senescence‐associated features, including the senescence‐associated secretory phenotype (SASP), wherein senescent chondrocytes secrete proinflammatory cytokines (IL‐6, TNF‐*α*) and matrix‐degrading enzymes (MMP3, MMP13) implicated in cartilage degeneration [34]. By partially mitigating these senescence‐related responses, p21 suppression may help reduce local inflammation and matrix breakdown, thereby creating a microenvironment more conducive to tissue repair. However, in the current study, direct evidence regarding the impact on SASP has not been provided and therefore additional research into this area is required.

A particularly important finding is the equivalent efficacy in both immunocompromised (B6.Cg‐Prkdc^scid^/SzJ) and immunocompetent (C57BL6) animals, demonstrating that humoral or cell‐mediated immune responses against lentiviral vectors do not impair local therapeutic efficacy. This robustness across immune backgrounds strongly supports clinical translation, as it suggests the approach is viable in immunocompetent patients without requiring immunosuppression.

It is important to acknowledge that p21 knockdown likely promotes cartilage repair through both canonical nuclear and noncanonical cytoplasmic mechanisms. Figure 4 demonstrates that cartilage injury induces p21 expression in both nuclear and cytoplasmic compartments of articular chondrocytes, which is effectively suppressed by lentiviral shRNA in both locations. While nuclear p21 primarily inhibits cyclin‐dependent kinases to enforce G1/S cell cycle arrest, as evidenced by increased G2/M‐phase cells following knockdown, cytoplasmic p21 exerts cyclin‐independent effects by stabilizing IκB*α* and suppressing NF‐κB transcriptional activity. This dual‐compartment reduction likely contributes to repair by simultaneously enhancing chondrocyte proliferation and attenuating injury‐induced inflammatory signaling, broadening p21's therapeutic relevance beyond cell cycle regulation alone.

### 4.3 Injury‐Dependent Systemic Dissemination and Safety

A striking finding is the injury‐dependent increase in hepatic lentiviral transduction. Uninjured animals showed minimal off‐target hepatic transduction, demonstrating that intact joints effectively compartmentalize intraarticular injections. In contrast, injured animals, which not only had a focal cartilage injury but also the surgery required to complete the injury, showed dramatically increased transduction (~30% in immunocompromised, ~15% in immunocompetent). This ~30‐fold increase in immunocompromised mice suggests that intact adaptive immunity provided partial containment of systemic dissemination, as evidenced by 2‐fold lower hepatic transduction in immunocompetent versus immunocompromised animals. Importantly, despite 2‐fold higher hepatic transduction in immunocompromised mice, cartilage repair was equivalent between strains, indicating that systemic transduction does not contribute to therapeutic benefit.

Several mechanisms likely explain injury‐dependent systemic dissemination. The surgical defect penetrating into subchondral bone creates a wound environment with neovascularization and increased vascular permeability, compromising the normally restrictive blood‐joint interface. Additionally, injury‐activated lymphatic drainage and recruitment of immune cells (macrophages, dendritic cells) could mobilize lentiviral particles to draining lymph nodes and distal sites. The 3‐day post‐injury injection timing may occur during peak barrier compromise; future studies varying injection timing would clarify the optimal window for efficacy and safety.

A paradoxical finding is that p21 expression increased in liver despite increased lentiviral transduction. While overt signs of liver injury were not observed, it is possible that the cartilage/joint injury could have resulted in a systemic injury‐induced upregulation of hepatic p21 through inflammatory cytokine signaling (elevated IL‐6, TNF‐*α*, IL‐1*β*), which overrides local lentiviral‐mediated knockdown [35]. This dissociation could demonstrate that systemic physiological stress responses to remote trauma independently activate p21 in distal tissues. It is also important to recognize that histological assessment revealed normal hepatic architecture across all groups with no inflammatory infiltration, hepatocellular necrosis, or apoptosis, despite hepatic transduction rates of 15%–30%, levels exceeding those in typical gene therapy studies. This absence of hepatotoxicity is reassuring for clinical safety and supports the favorable properties of nonreplicating lentiviral vectors.

### 4.4 Comparison to Alternatives and Future Directions

The efficacy achieved represents advancement compared to current surgical therapies. Microfracture produces fibrocartilage with poor durability; ACI causes donor site morbidity and involves chondrocyte de‐differentiation during ex vivo expansion [4]. This p21 knockdown approach works with resident chondrocytes, avoids cellular manipulation, and provides sustained transgene expression through chromosomal integration, potentially critical given extended cartilage healing timelines.

Important limitations include the short 4‐week observation period, lack of biomechanical testing (as histology alone does not confirm functional mechanical recovery), and incomplete molecular characterization. Markers of fibrocartilage formation were not examined; however, the presence of a collagen Type II matrix is encouraging and suggests maintenance of a hyaline‐like cartilage phenotype. Additionally, SASP markers (IL‐6, TNF‐*α*, MMP13) were not measured, leaving the senescence‐suppression hypothesis only partially tested. Future studies should incorporate extended timepoints, biomechanical assessment, transcriptomic profiling, evaluation of combination strategies with scaffolds or growth factors, and validation in larger animal models.

## 5 Conclusion

This study demonstrates that targeted p21 knockdown via lentiviral shRNA delivery effectively promotes cartilage regeneration in focal cartilage defects with robust efficacy across immunocompromised and immunocompetent hosts. High transduction efficiency, substantial p21 knockdown, and significant correlation between p21 levels and repair outcomes provide mechanistic validation. While injury increases systemic viral dissemination, the absence of hepatotoxicity and maintained efficacy in immunocompetent animals support clinical feasibility. These findings provide evidence for targeting p21 as a therapeutic strategy for cartilage repair and provide rationale for further development.

## Author Contributions

L.L., A.T.S., and R.J.K. conceived, designed, and oversaw experiments with scientific assistance from A.A.A. and N.O.V. L.L. performed all experiments, analyzed data, and wrote the manuscript. A.A.A. assisted with data collection. R.J.K. assisted with data interpretation. All authors were involved in editing the manuscript, and all authors have approved the final version for submission.

## Funding

This work was supported by U of C | CSM | McCaig Institute for Bone and Joint Health, Cumming School of Medicine, University of Calgary (McCaig Institute), PDF Fellowship, Canada Foundation for Innovation (CFI), JELF, Calgary Foundation (CF), Grace Glaum Professorship, and T ‐ Technologies Grundlagenforschung EU, n/a.

## Conflicts of Interest

A.T.S. is the founder and equity holder in T‐Technologies Grundlagenforschung e.U. N.O.V. is an employee of T‐Technologies Grundlagenforschung e.U.

## Supporting information


**Figure S1:** Validation of *p21* knockdown. Flowchart of the experimental design (A). FACS was employed to identify and purify cells expressing tdTomato (B‐D). qPCR was performed on the tdTomato positive cells expressing the nonsense or *p21* shRNA (F‐K). Cell cycle analysis was undertaken by flow cytometry and the percentage of cells within G1 and G2/M are shown. Six technical replicates were used per group, and one‐way ANOVA was used to determine significance. *p* was set to 0.05.
**Figure S2:** Cartilage repair in immunocompetent mice treated with lentivirus containing *p21* shRNA. Histological sections from uninjured, nonsense and *p21* shRNA treated mice were stained with antibodies against Col2, p16, and IL‐6 (A). The mean fluorescent intensity (MFI) of Col2 staining was quantified (B) along with the percentage of cells positive for p16 (C) or IL‐6 (D). Ten biological/mice replicates were used per group (5 M, 5F) and one‐way ANOVA was used to determine significance. *p* was set to 0.05. Scale bars equal 35 μm.

## Data Availability

The data that support the findings of this study are available in the methods and/or Supporting Information of this article.

## References

[1] A. O. Masson and R. J. Krawetz , “Understanding Cartilage Protection in OA and Injury: A Spectrum of Possibilities,” BMC Musculoskeletal Disorders 21 (2020): 432. 32620156 10.1186/s12891-020-03363-6 PMC7334861

[2] W. Hunter , “VI. Of the Structure and Diseases of Articulating Cartilages,” Philosophical Transactions of the Royal Society of London 42 (1743): 514–521.

[3] D. Primorac , V. Molnar , E. Rod , et al., “Knee Osteoarthritis: A Review of Pathogenesis and State‐Of‐The‐Art Non‐Operative Therapeutic Considerations,” Genes (Basel) 11 (2020): 1–35. 10.3390/genes11080854 PMC7464436 32722615

[4] J. C. Mora , R. Przkora , and Y. Cruz‐Almeida , “Knee Osteoarthritis: Pathophysiology and Current Treatment Modalities,” Journal of Pain Research 11 (2018): 2189–2196. 30323653 10.2147/JPR.S154002 PMC6179584

[5] M. J. Kraeutler , J. W. Belk , J. M. Purcell , and E. C. McCarty , “Microfracture Versus Autologous Chondrocyte Implantation for Articular Cartilage Lesions in the Knee: A Systematic Review of 5‐Year Outcomes,” American Journal of Sports Medicine 46 (2018): 995–999. 28423287 10.1177/0363546517701912

[6] E. H. Kim , S. Jeon , J. Park , et al., “Progressing Future Osteoarthritis Treatment Toward Precision Medicine: Integrating Regenerative Medicine, Gene Therapy and Circadian Biology,” Experimental & Molecular Medicine 57 (2025): 1133–1142. 40588525 10.1038/s12276-025-01481-6 PMC12229678

[7] J. L. Koh , K. C. Jacob , R. Kulkarni , Z. Vasilion , and F. M. L. Amirouche , “Consequences of Progressive Full‐Thickness Focal Chondral Defects Involving the Medial and Lateral Femoral Condyles After Meniscectomy: A Biomechanical Study Using a Goat Model,” Orthopaedic Journal of Sports Medicine 10 (2022): 23259671221078600. 10.1177/23259671221078598 PMC8958688 35356308

[8] G. Merkely , J. Ackermann , and C. Lattermann , “Articular Cartilage Defects: Incidence, Diagnosis, and Natural History,” Oper. Tech. Sports Med 26 (2018): 156–161.

[9] Q. Yao , X. Wu , C. Tao , et al., “Osteoarthritis: Pathogenic Signaling Pathways and Therapeutic Targets,” Signal Transduction and Targeted Therapy 8 (2023): 56. 36737426 10.1038/s41392-023-01330-w PMC9898571

[10] B. Fu , J. Shen , X. Zou , et al., “Matrix Stiffening Promotes Chondrocyte Senescence and the Osteoarthritis Development Through Downregulating HDAC3,” Bone Research 12 (2024): 32. 38789434 10.1038/s41413-024-00333-9 PMC11126418

[11] B. Wang , J. Han , J. H. Elisseeff , and M. Demaria , “The Senescence‐Associated Secretory Phenotype and Its Physiological and Pathological Implications,” Nature Reviews Molecular Cell Biology 25 (2024): 958–978. 38654098 10.1038/s41580-024-00727-x

[12] B. O. Diekman and R. F. Loeser , “Aging and the Emerging Role of Cellular Senescence in Osteoarthritis,” Osteoarthritis and Cartilage 32 (2024): 365–371. 38049031 10.1016/j.joca.2023.11.018 PMC10984800

[13] Z. Han , K. Wang , S. Ding , and M. Zhang , “Cross‐Talk of Inflammation and Cellular Senescence: A New Insight Into the Occurrence and Progression of Osteoarthritis,” Bone Research 12 (2024): 69. 39627227 10.1038/s41413-024-00375-z PMC11615234

[14] O. Cazzalini , A. I. Scovassi , M. Savio , L. A. Stivala , and E. Prosperi , “Multiple Roles of the Cell Cycle Inhibitor p21CDKN1A in the DNA Damage Response,” Mutation Research, Reviews in Mutation Research 704 (2010): 12–20. 10.1016/j.mrrev.2010.01.009 20096807

[15] C. L. Jablonski , B. A. Besler , J. Ali , and R. J. Krawetz , “p21−/− Mice Exhibit Spontaneous Articular Cartilage Regeneration Post‐Injury,” Cartilage 13 (2021): 1947603519876348. 10.1177/1947603519876348 PMC8804758 31556320

[16] K. Ibaraki , S. Hayashi , N. Kanzaki , et al., “Deletion of p21 Expression Accelerates Cartilage Tissue Repair via Chondrocyte Proliferation,” Molecular Medicine Reports 21 (2020): 2236–2242. 32186772 10.3892/mmr.2020.11028

[17] S. D'Costa , M. J. Rich , and B. O. Diekman , “Engineered Cartilage From Human Chondrocytes With Homozygous Knockout of Cell Cycle Inhibitor p21,” Tissue Engineering. Part A 26 (2020): 441–449. 31642391 10.1089/ten.TEA.2019.0214

[18] B. O. Diekman , P. I. Thakore , S. K. O'Connor , et al., “Knockdown of the Cell Cycle Inhibitor p21 Enhances Cartilage Formation by Induced Pluripotent Stem Cells,” Tissue Engineering. Part A 21 (2015): 1261–1274. 25517798 10.1089/ten.tea.2014.0240 PMC4394871

[19] V. Dexheimer , S. Frank , and W. Richter , “Proliferation as a Requirement for In Vitro Chondrogenesis of Human Mesenchymal Stem Cells,” Stem Cells and Development 21 (2012): 2160–2169. 22229819 10.1089/scd.2011.0670 PMC3411365

[20] T.‐L. Yew , F.‐Y. Chiu , C.‐C. Tsai , et al., “Knockdown of p21(Cip1/Waf1) Enhances Proliferation, the Expression of Stemness Markers, and Osteogenic Potential in Human Mesenchymal Stem Cells,” Aging Cell 10 (2011): 349–361. 21342417 10.1111/j.1474-9726.2011.00676.x

[21] A. O. Masson , R. Hess , K. O'Brien , et al., “Increased Levels of p21((CIP1/WAF1)) Correlate With Decreased Chondrogenic Differentiation Potential in Synovial Membrane Progenitor Cells,” Mechanisms of Ageing and Development 149 (2015): 31–40. 25987237 10.1016/j.mad.2015.05.005

[22] K. L. Bertram , N. Narendran , P. Tailor , et al., “17‐DMAG Regulates p21 Expression to Induce Chondrogenesis In Vitro and In Vivo,” Disease Models & Mechanisms 11 (2018): dmm033662. 30305302 10.1242/dmm.033662 PMC6215425

[23] P. Sheng , K. A. Flood , and M. Xie , “Short Hairpin RNAs for Strand‐Specific Small Interfering RNA Production,” Frontiers in Bioengineering and Biotechnology 8 (2020): 573098. 10.3389/fbioe.2020.00940 PMC7427337 32850763

[24] E. Fang , G. He , Y. Chang , Q. He , P. Chen , and K. Hu , “Application Advances of Lentiviral Vectors: From Gene Therapy to Vaccine Development,” Molecular Biotechnology 2025 (2025): 1–17. 10.1007/s12033-025-01472-y 40617903

[25] J. Mak , C. L. Jablonski , C. A. Leonard , et al., “Intra‐Articular Injection of Synovial Mesenchymal Stem Cells Improves Cartilage Repair in a Mouse Injury Model,” Science Reporter 6 (2016): 23076. 10.1038/srep23076 PMC4794799 26983696

[26] C. L. Jablonski , C. Leonard , P. Salo , and R. J. Krawetz , “CCL2 but Not CCR2 Is Required for Spontaneous Articular Cartilage Regeneration Post‐Injury,” Journal of Orthopaedic Research 37 (2019): 2561–2574. 31424112 10.1002/jor.24444

[27] J. Fitzgerald , C. Rich , D. Burkhardt , J. Allen , A. S. Herzka , and C. B. Little , “Evidence for Articular Cartilage Regeneration in MRL/MpJ Mice,” Osteoarthritis and Cartilage 16 (2008): 1319–1326. 18455447 10.1016/j.joca.2008.03.014

[28] N. M. Eltawil , C. De Bari , P. Achan , C. Pitzalis , and F. Dell'accio , “A Novel In Vivo Murine Model of Cartilage Regeneration. Age and Strain‐Dependent Outcome After Joint Surface Injury,” Osteoarthritis and Cartilage 17 (2009): 695–704. 19070514 10.1016/j.joca.2008.11.003 PMC2706394

[29] L. Ferrie , P. Premnath , A. Olsen , et al., “Exogenously Delivered iPSCs Disrupt the Natural Repair Response of Endogenous MPCs After Bone Injury,” Science Reporter 13 (2023): 9378. 10.1038/s41598-023-36609-z PMC10256810 37296277

[30] C. L. Jablonski , D. Modrcin , J. Cobb , D. M. McCafferty , P. T. Salo , and R. J. Krawetz , “Prx1+ Progenitors Give Rise to New Articular Cartilage When Conditions Are Permissive for Endogenous Regeneration,” npj Regenerative Medicine 10 (2025): 38. 40783399 10.1038/s41536-025-00425-y PMC12335530

[31] K. Bedelbaeva , A. Snyder , D. Gourevitch , et al., “Lack of p21 Expression Links Cell Cycle Control and Appendage Regeneration in Mice,” Proceedings of the National Academy of Sciences of the United States of America 107 (2010): 5845–5850. 20231440 10.1073/pnas.1000830107 PMC2851923

[32] A. Annoni , S. Gregori , L. Naldini , and A. Cantore , “Modulation of Immune Responses in Lentiviral Vector‐Mediated Gene Transfer,” Cellular Immunology 342 (2019): 103802. 29735164 10.1016/j.cellimm.2018.04.012 PMC6695505

[33] P. Hu , Y. Hao , W. Tang , G. H. Diering , F. Zou , and T. Kafri , “Analysis of Hepatic Lentiviral Vector Transduction; Implications for Preclinical Studies and Clinical Gene Therapy Protocols,” (2024) bioRxiv 2024.08.20.608805. 10.3390/v17020276 PMC11861806 40007031

[34] X. Zhao , J. Lin , F. Liu , et al., “Targeting p21‐Positive Senescent Chondrocytes via IL‐6R/JAK2 Inhibition to Alleviate Osteoarthritis,” Advanced Science 12 (2025): 2410795. 39853717 10.1002/advs.202410795 PMC11923994

[35] J. Yi , H. Zhang , F. Bao , et al., “A Pathological Joint–Liver Axis Mediated by Matrikine‐Activated CD4+ T Cells,” Signal Transduction and Targeted Therapy 9 (2024): 109. 38714712 10.1038/s41392-024-01819-y PMC11076293

