# Supplementary material for: p21 Knockdown as a Therapeutic Strategy for Focal Cartilage Injury Repair
Source: FASEB J. 2026 Apr 20;40:e71814. doi: 10.1096/fj.202600387RRR (PMC13094461; doi:10.1096/fj.202600387RRR)
Supplement: Supplementary file 1 — Figure S1: Validation of p21 knockdown. Flowchart of the experimental design (A). FACS was employed to identify and purify cells expressing tdTomato (B‐D). qPCR was performed on the tdTomato positive cells expressing the nonsense or p21 shRNA (F‐K). Cell cycle analysis was undertaken by flow cytometry and the percentage of cells within G1 and G2/M are shown. Six technical replicates were used per group, and one‐way ANOVA was used to determine significance. p was set to 0.05. Figure S2: Cartilage repair in immunocompetent mice treated with lentivirus containing p21 shRNA. Histological sections from uninjured, nonsense and p21 shRNA treated mice were stained with antibodies against Col2, p16, and IL‐6 (A). The mean fluorescent intensity (MFI) of Col2 staining was quantified (B) along with the percentage of cells positive for p16 (C) or IL‐6 (D). Ten biological/mice replicates were used per group (5 M, 5F) and one‐way ANOVA was used to determine significance. p was set to 0.05. Scale bars equal 35 μm. [file FSB2-40-e71814-s001.docx]

**
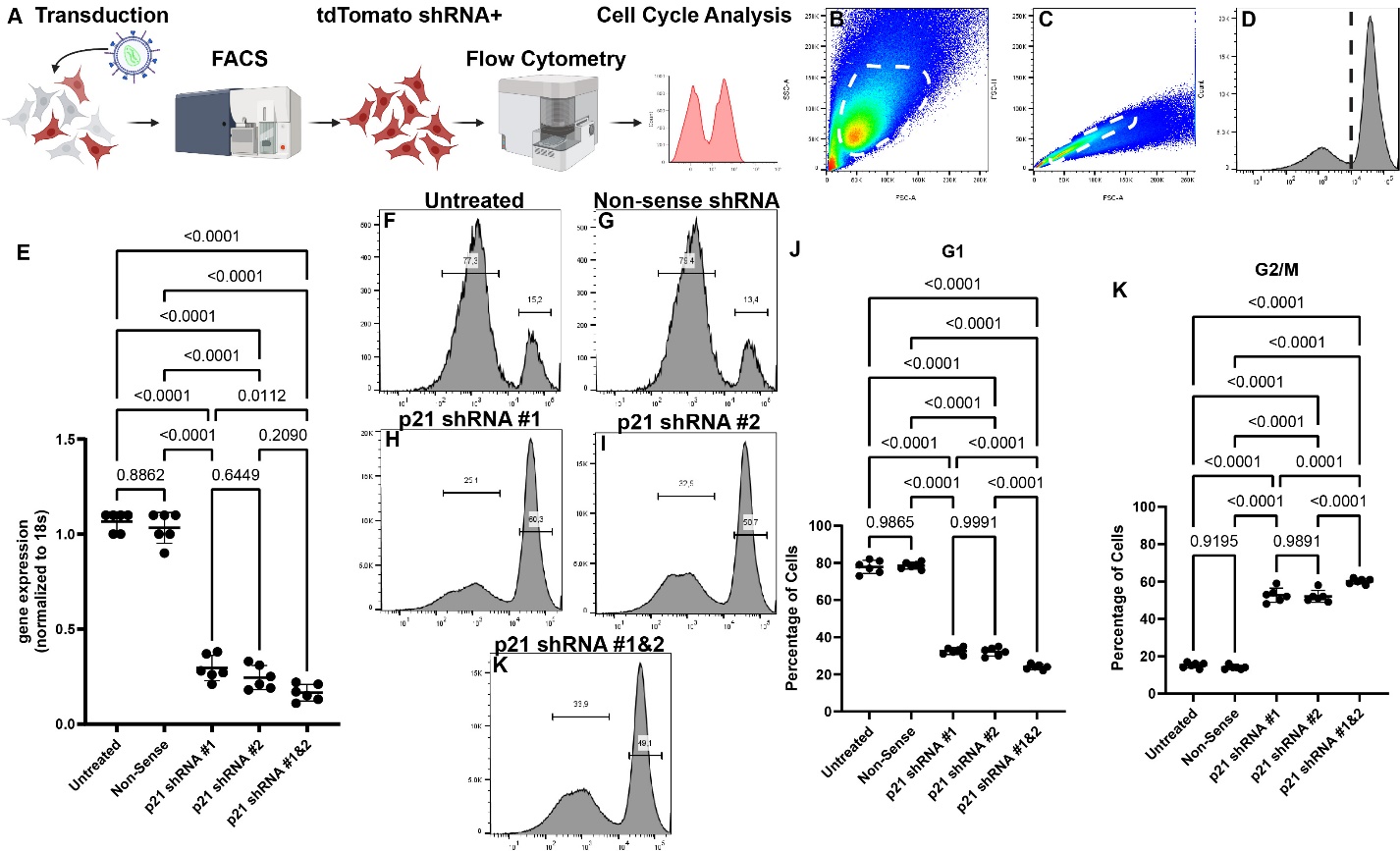
**

**Figure S1. Validation of *p21* knockdown**. Flowchart of the experimental design (A). FACS was employed to identify and purify cells expressing tdTomato (B-D). qPCR was performed on the tdTomato positive cells expressing the non-sense or *p21* shRNA (F-K). Cell cycle analysis was undertaken by flow cytometry and the percentage of cells within G1 and G2/M are shown. Six technical replicates were used per group, and 1-way ANOVA was used to determine significance. p was set to 0.05.


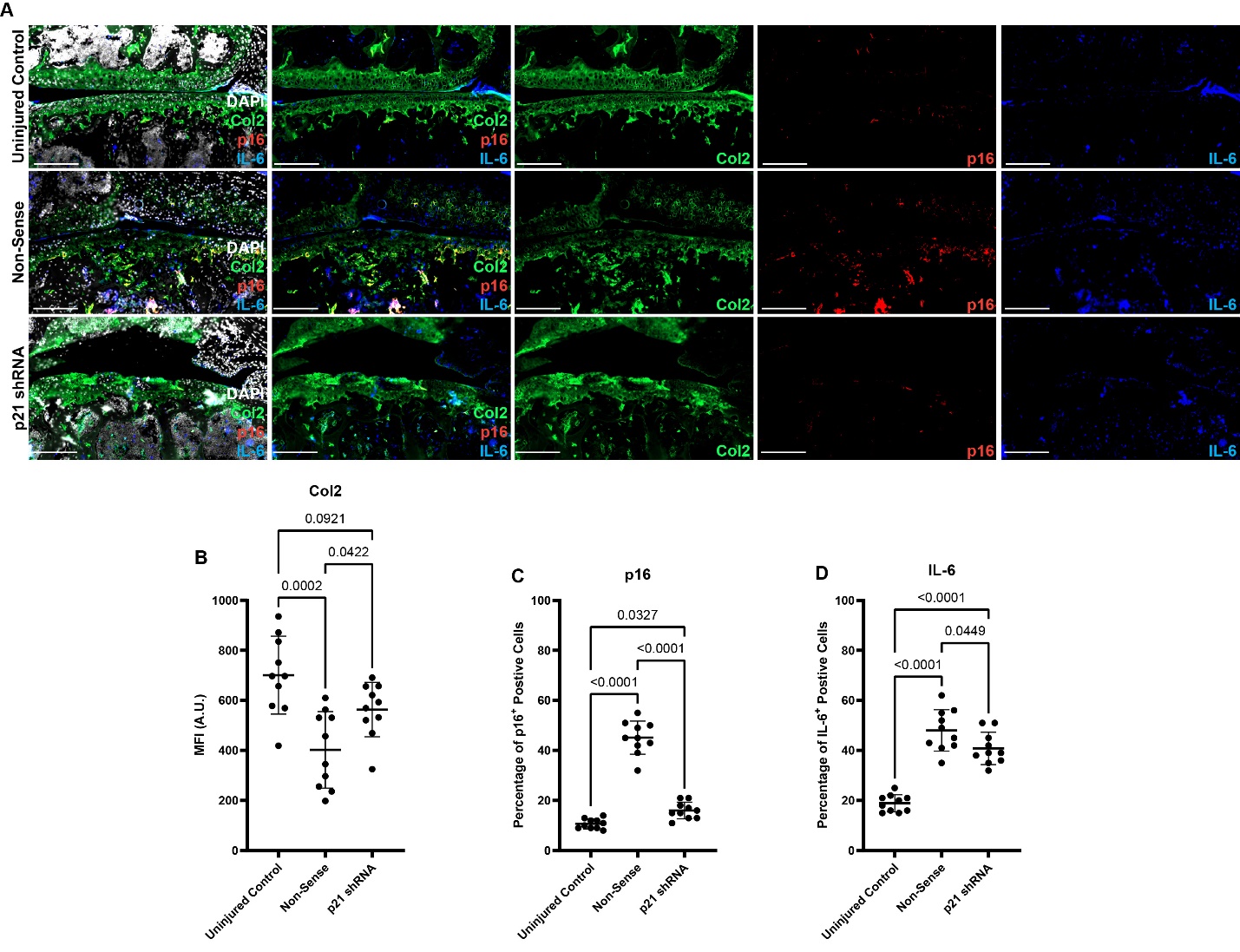


**Figure S2. Cartilage repair in immunocompetent mice treated with lentivirus containing *p21* shRNA**. Histological sections from uninjured, non-sense and *p21* shRNA treated mice were stained with antibodies against Col2, p16 and IL-6 (A). The mean fluorescent intensity (MFI) of Col2 staining was quantified (B) along with the percentage of cells positive for p16 (C) or IL-6 (D). Ten biological/mice replicates were used per group (5M, 5F) and 1-way ANOVA was used to determine significance. p was set to 0.05. Scale bars equal 35µm.
